# Supplementary material for: Benefit versus risk of chromosomal microarray analysis performed in pregnancies with normal and positive prenatal screening results: A retrospective study
Source: PLoS One. 2021 Apr 26;16(4):e0250734. doi: 10.1371/journal.pone.0250734 (PMC8075189; doi:10.1371/journal.pone.0250734)
Supplement: S1 Table — Almost half (48.22%) of the indications for amniocentesis in the study cohort were non-medical: 30.07% of women underwent amniocentesis due to advanced maternal age while 18.14% had no indication for this procedure. The most common reason for amniocentesis for a medical indication was abnormal prenatal screening results (26.04%). (DOCX) [file pone.0250734.s001.docx]

**S1 Table. Indications for amniocentesis**

| **Total**  **N=30,830**  **n (%)**  **n**  **n (%)** | **Indication** |
| --- | --- |
| **Non-medical indications (maternal choice)** | |
| 9,270 (30.07) | - **Advanced maternal age** |
| 5,591 (18.14) | - **Without indication** |
| 3 (0.01) | - **Dilution of one embryo (out of two embryos)** |
| **Medical indications** | |
| 8,031 (26.04) | - **Abnormal screening test results** |
| 619 (2.00) | - **Cytomegalovirus** |
| 590 (1.91) | - **Abnormal ultrasound organ scan results** |
| 231 (0.75) | - **Genetic disease carrier** |
| 223 (0.72) | - **Family history of congenital abnormalities** |
| 221 (0.72) | - **History of a baby born with abnormal chromosomes** |
| 171 (0.55) | - **History of a structurally abnormal baby** |
| 56 (0.18) | - **Medications that may induce chromosomal changes^a^** |
| 44 (0.14) | - **Previous pregnancy with abnormal karyotype/chromosomal microarray analysis results detected in the foetus** |
| 33 (0.11) | - **Abnormal non-invasive prenatal testing results** |
| 30 (0.10) | - **Parents are translocation carriers** |
| 18 (0.06) | - **Suspected toxoplasma infection** |
| 16 (0.05) | - **High human chorionic gonadotropin** |
| 5 (0.02) | - **Suspected Turner syndrome by non-invasive prenatal testing** |
| 5 (0.02) | - **Low pregnancy-associated plasma protein-A** |
| 2 (0.01) | - **Low estriol** |
| 1 (0.003) | - **Abnormal chorionic villus sampling results** |
| 5670 (18.39) | - **No data on indication in medical file** |

^a^Medications that may induce chromosomal changes include colchicine, podophyllotoxin, 6-mercaptopurine, 5-fluorouracil, azathioprine, and propylthiouracil.
