# Supplementary material for: Benefit versus risk of chromosomal microarray analysis performed in pregnancies with normal and positive prenatal screening results: A retrospective study
Source: PLoS One. 2021 Apr 26;16(4):e0250734. doi: 10.1371/journal.pone.0250734 (PMC8075189; doi:10.1371/journal.pone.0250734)
Supplement: S2 Table — There were no differences between maternal age groups, except for higher weight on the day of the procedure and a higher prevalence of pregnancies that started out with multiple embryos, and fertility treatments in women ≥35 years compared to women <35 years. Gestational age on the day of the procedure was also significantly higher in women <35 years compared to women ≥35 years; however, it was not clinically significant. Paternal age, the number of pregnancies, and the number of deliveries were higher among women ≥35 years compared to women <35 years, as they are directly related to maternal age. (DOCX) [file pone.0250734.s002.docx]

**S2 Table. Characteristics of pregnancies with normal karyotype and normal prenatal screening results for which amniocenteses were performed for CMA**

| **Characteristic** | **<35 years**  **N=1,702** | **≥35 years**  **N=2,472** | **P-value** |
| --- | --- | --- | --- |
| **Socio-demographic data** | | | |
| - **Maternal age (years), mean±SD** | 31.6±2.0 | 37.9±2.4 | <0.0001 |
| - **Gestational age (weeks), mean±SD^a^** | 18.5±1·3 | 18.3±1.2 | <0.0001 |
| - **Weight (kg), mean±SD^a^** | 62.4±9.8 | 65.9±10.9 | <0.0001 |
| - **Paternal age (years), mean ± SD** | 33.9±3.9 | 39.5±8.2 | <0.0001 |
| - **Consanguinity between parents, n/N (%)** | 2/1,695 (0.1) | 3/2,348 (0.1) | >0.99 |
| **Obstetrics history** | | | |
| - **Number of pregnancies, median (IQR)^b^** | 2.0 (1.0-2.0) | 3.0 (2.0-4.0) | <0.0001 |
| - **Number of deliveries, median (IQR)** | 1.0 (0.0-1.0) | 2.0 (1.0-2.0) | <0.0001 |
| **Obstetric complications during the current pregnancy** | | | |
| - **Vaginal bleeding, n/N (%)** | 67/1,702 (3.9) | 117/2,472 (4.7) | 0.28 |
| - **Cervical incompetence with/without cerclage, n/N (%)** | 4/1,702 (0.2) | 8/2,472 (0.3) | 0.77 |
| - **Pregnancy started out with multiple embryos, n/N (%)** | 13/1,702 (0.8) | 57/2,472 (2.3) | <0.0001 |
| - **Fertility treatment, n/N (%)** | 78/1,702 (4.6) | 231/2,472 (9.3) | <0.0001 |
| - **Overt diabetes, n/N (%)** | 7/1,702 (0.4) | 12/2,472 (0.5) | 0.82 |
| - **Pregnancy-induced diabetes, n/N (%)** | 4/1,702 (0.2) | 14/2,472 (0.6) | 0.15 |
| - **Essential hypertension, n/N (%)** | 2/1,702 (0.1) | 1/2,472 (0.0) | 0.57 |
| - **Pregnancy-induced hypertension, n/N (%)** | 1/1,702 (0.1) | 0/2,472 (0.0) | 0.41 |

Abbreviations: CMA, chromosomal microarray analysis; IQR, interquartile range; SD, standard deviation

^a^Gestational age and weight on day of procedure

^b^Including the current pregnancy
